# Evidence of Paraoxonases 1, 2, and 3 Expression in Human Ovarian Granulosa Cells

**DOI:** 10.3390/antiox10101504

**Published:** 2021-09-22

**Authors:** Irantzu Pérez-Ruiz, José-Ignacio Ruiz-Sanz, María-Luisa Hérnandez, Rosaura Navarro, Marcos Ferrando, Zaloa Larreategui, María-Begoña Ruiz-Larrea

**Affiliations:** 1 Free Radicals and Oxidative Stress (FROS) Research Group of the Department of Physiology, Medicine and Nursing School, University of the Basque Country UPV/EHU, 48940 Leioa, Spain; irantzuperez0@gmail.com (I.P.-R.); joseignacio.ruizs@ehu.eus (J.-I.R.-S.); luisa.hernandez@ehu.eus (M.-L.H.); rosaura.navarro@ehu.eus (R.N.); 2 BioCruces Health Research Institute, Plaza de Cruces s/n, 48903 Barakaldo, Spain; 3 Valencian Institute of Infertility (IVI-RMA)-Bilbao, 48940 Leioa, Spain; marcos.ferrando@ivirma.com (M.F.); zaloa.larreategui@ivirma.com (Z.L.)

**Keywords:** assisted reproduction, ovary, granulosa cells, paraoxonase, oxidative stress, antioxidant

## Abstract

Increasing evidence suggests that the antioxidant paraoxonase proteins, PON1, PON2, and PON3, have a role in reproduction and may be synthesized by ovarian cells. The aim of this work was to investigate whether human ovarian granulosa cells (GC) express paraoxonases 1, 2, and 3 (PON1, PON2, and PON3) at both the transcriptional and protein levels. Cells were purified from follicle samples of women undergoing ovarian stimulation at oocyte retrieval. We analyzed mRNA by polymerase chain reaction using specific primers for the different variants and quantified the proteins by Western blot using commercially available human recombinant PON proteins as standards. The protein subcellular distribution was determined by immunofluorescence and confocal microscopy and the cell cycles by flow cytometry. Thymidine was used for cellular synchronization at G1/S. Human hepatoma HepG2 and immortalized granulosa COV434 cell lines were used to optimize methodologies. mRNAs from PON1, the two variants of PON2, and PON3 were detected in GC. The cells actively secreted PON1 and PON3, as evidenced by the protein detection in the incubation medium. PON1 and PON3 were mainly distributed in the cytoplasm and notably in the nucleus, while PON2 colocalized with mitochondria. Subcellular nucleo-cytoplasmic distribution of PON1 was associated with the cell cycle. This is the first evidence describing the presence of mRNAs and proteins of the three members of the PON family in human ovarian GC. This study provides the basis of further research to understand the role of these proteins in GC, which will contribute to a better understanding of the reproduction process.

## 1. Introduction

The use of assisted reproduction techniques has increased exponentially during recent years in developed societies in association with fertility decline. Although free radicals and reactive oxygen species (ROS) are necessary in different steps during female reproduction, high concentrations of these species are associated with reproductive diseases and infertility [1]. To maintain these species at physiologically relevant stable concentrations, both enzymatic and non-enzymatic antioxidants act coordinately in the body. Among the antioxidant enzymes are paraoxonases (PONs). The human paraoxonase gene family consists of three members: PON1 (ID HGNC:9204), PON2 (ID HGNC:9205), and PON3 (ID HGNC:9206), which are located adjacently in a cluster on the long arm of chromosome 7 [2,3,4]. The PON1 and PON3 proteins are predominantly synthesized in the liver, secreted to the bloodstream, and transported by lipoproteins, mainly associated with high-density lipoproteins (HDL) [5]. In contrast, PON2 is intracellularly located in a wide variety of tissues and cells [3,6].

Most of the references regarding the clinical implications of PON proteins deal with cardiovascular diseases. In the blood, PON1 and PON3 exert antioxidant actions against the oxidative modifications of low-density lipoproteins, thus protecting against atherosclerosis [5,7,8]. However, the role of PON proteins in infertility is not clearly defined. Several works have reported the implication of paraoxonase activities in seminal plasma and sperm parameters in subfertile men [9,10,11]. Regarding female infertility, significant changes in serum paraoxonase activities were associated with polycystic ovary syndrome (PCOS) [12,13]. In previous work, we reported that the follicular fluid of developing follicles from patients undergoing ovarian stimulation presented catalytic activities of PON1, PON2, and PON3, and these activities increased as the follicle grew. We also showed higher PON3 activity in follicular fluid from donors than from unfertile patients [14]. Follicular fluid is constituted by elements from the blood that are small enough to pass the blood/follicle barrier, HDL among them, and other components that are locally secreted by the ovary cells. The comparisons of the mass quantity and activity of the extracellular PON1 and PON3 proteins between serum and follicular fluid suggested that their activities in follicular fluid were not simply due to a higher HDL inflow from the serum [14].

Although the presence of secretory paraoxonases in follicular fluid has been described, there are no data on their local synthesis by ovarian cells. In this study, we addressed the expressions of PON1, PON2, and PON3 at the transcriptional and translational levels and their subcellular location in the granulosa cells from women subjected to ovarian stimulation.

## 2. Materials and Methods

### 2.1. Human Samples

Samples were collected from 20 female patients of the IVI-RMA assisted reproduction clinic in Bilbao. Women underwent controlled ovarian stimulation according to the protocol of the clinic [15]. At the time of oocyte retrieval, samples were obtained by follicular puncture. The oocyte was separated from the follicular fluid and the rest of the ovary cells. This biological material, which constitutes medical waste, was the starting biological material used to purify granulosa cells. The Ethics Committee for Research involving Human Subjects of the University (CEISH) approved the protocol (M30_2015_187_RUIZ LARREA, 28 October 2015). All women who agreed to participate in the study signed an informed consent form.

### 2.2. Purification and Culture of Granulosa Cells

Granulosa cells were isolated using Percoll solutions (40% and 60%) prepared in McCoy’s medium (Sigma-Aldrich, St. Louis, MO, USA) supplemented with 10% heat-inactivated fetal bovine serum (FBS). The biological samples were centrifuged at 600× *g* for 10 min at room temperature. The cellular pellet was resuspended in McCoy’s medium, and 1 mL of the mixture was carefully layered on polystyrene tubes containing Percoll gradient (2:2:1, *v/v/v*). The tubes were centrifuged at 600× *g* for 20 min at room temperature. After centrifugation, the cells were collected from the interface. This process was repeated once. The isolated cells were washed from Percoll by differential centrifugation. Finally, granulosa cells were used immediately. The purified cells were cultured in gelatin-coated Petri dishes in McCoy’s medium with 10% fetal bovine serum (FBS), to which apotransferrin (5 mg/mL), Δ^4^-androstenedione (0.1 μM), HNaCO_3_ (2.2 g/L), and HEPES (20 mM) were added. All these materials were purchased from Sigma-Aldrich (St. Louis, MO, USA). The culture was maintained in the incubator (5% CO_2_) at 37 °C for 48 h. Samples were taken for cell count and viability testing using Trypan blue, and processed for RNA extraction, Western blot, and immunocytochemical analysis.

### 2.3. Culture of Cell Lines

The human ovary COV434 granulosa cells (Sigma-Aldrich, St. Louis, MO, USA) and human HepG2 hepatoma cell line (ATCC, Manassas, VA, USA) were seeded in Dulbecco’s modified Eagle medium and Eagle’s minimum essential medium (Sigma-Aldrich, St. Louis, MO, USA), respectively. Both media were supplemented with 10% FBS, 2 mM L-glutamine, 0.1 mg/mL streptomycin, and 100 U/mL penicillin. Cell lines were cultured (5% CO_2_) at 37 °C in six-well plates.

### 2.4. RNA Processing and Analysis by PCR

TRIzol reagent was used for the extraction of total RNA following the manufacturer’s protocol (Thermo Fisher Scientific, Waltham, MA, USA). RNA concentration was determined spectrophotometrically at 260 nm (NanoDrop, Thermo Fisher Scientific, Waltham, MA, USA) and the purity was assessed by the ratio of the absorbances at 260 and 280 nm. RNA was treated with extension-grade DNase I (Thermo Fisher Scientific, Waltham, MA, USA) to eliminate possible DNA contamination of the sample according to the manufacturer’s instructions. The cDNA used in the PCR assays was obtained from 1.5 µg of RNA by the retrotranscription reaction using the Superscript III First-Strand Synthesis System for RT-PCR kit (Thermo Fisher Scientific, Waltham, MA, USA) according to the manufacturer’s instructions. The final mix with amplified cDNA was diluted up to 100 µL.

PON1, PON2, and PON3 mRNAs were detected by conventional RT-PCR. The PCR was performed in a Biometra thermocycler using primers designed with the aid of the bioinformatics tool Primer-Blast (https://www.ncbi.nlm.nih.gov/tools/primer-blast/, date of access 21 September 2020) [16] (Table 1).

PON1 and PON2 PCRs were carried out in a final volume of 40 µL containing 1× Immobuffer, 2.5 mM Cl_2_Mg, 200 µM/nt dNTP mix, 5% DMSO, 0.5 µM of each primer, 1.25 U of IMMOLASE^TM^ DNA Polymerase (Bioline, Luckenwalde, Germany), and 5 µL of previously diluted cDNA. PON3 nested PCR was carried out under the same conditions but without DMSO. The final conditions of the PCRs are shown in Table 2.

The products resulting from amplification were separated by horizontal electrophoresis on 2% agarose gels and SYBR safe staining. The bands were visualized by optical densitometry using the Bio-Rad Molecular Imager FX system.

### 2.5. Protein Quantification by Western Blot

At the indicated times, the culture medium was removed (reserved for further analysis), and the cells were washed twice with cold phosphate-buffered saline (PBS). Cells were lysed on ice for 30 min with lysis buffer, consisting of 20 mM HEPES pH 7.5, 1 mM NaF, 10 mM EGTA, 40 mM ß-glycerophosphate, 1% NP-40, 2.5 mM MgCl_2_, 2 mM orthovanadate, and 1 mM dithiothreitol, to which 10 μL/mL of a protease inhibitor cocktail (Sigma-Aldrich, St. Louis, MO, USA) was added just before use. Cellular debris was removed by centrifugation at 10,000× *g* for 5 min at 4 °C, and the supernatant was used for analysis. Total protein was quantified by staining with Coomassie brilliant blue [17]. Western blot quantification of PON1, PON2, and PON3 was carried out according to Meijide et al. [14]. To detect secreted PON1 and PON3 in the incubation medium, the medium was first enriched in minority proteins using Bio-Rad’s Proteominer kit by affinity column chromatography, as described previously [14].

### 2.6. Immunocytochemical Analysis

Cells were cultured on round covers under the conditions described above. After 48 h of culture, cells were fixed with 3.7% formaldehyde for 15 min at room temperature. The cells were washed three times and permeabilized with 0.5% Triton X-100 for 5 min at room temperature, and blocked with 10% SBF for 1 h. Samples were incubated overnight at 4 °C with the primary monoclonal antibodies, anti-PON1 (1:100), anti-PON2 (1:100) (R & D systems, Minneapolis, MN, USA), and anti-PON3 (1:200, Abcam, Cambridge, UK). In addition, the samples were incubated with monoclonal antibody anti-ATP5a (1:1000, Abcam, Cambridge, UK) for mitochondria detection. For synchronization studies, samples incubated with anti-PON1 were used (Thermo Fisher Scientific, Waltham, MA, USA). After several washes with PBS, the secondary antibodies (Thermo Fisher Scientific, Waltham, MA, USA) were added at a 1:200 dilution. Anti-rat IgG conjugated with Alexa Fluor488 was added for the detection of PON1 and PON2, anti-rabbit IgG conjugated with Alexa Fluor488 for the detection of PON3, and anti-mouse IgG conjugated with Alexa Fluor633 for the detection of mitochondria. Anti-mouse IgG antibody conjugated with Alexa Fluor488 was used to detect PON1 in synchronization assays. The nuclei were counterstained with 5 µg/mL DAPI (Sigma-Aldrich, St. Louis, MO, USA). Finally, the samples were placed on a slide with glycergel mounting medium (Dako, Carpintería, CA, USA). The images were acquired using a ZEISS LSM 880 confocal microscope with Airyscan high-resolution mode at the Analytical Microscopy Service of the UPV/EHU. The images were processed using ImageJ-win32 software (https://imagej.nih.gov/ij/, date of access 21 September 2020).

### 2.7. Cellular Synchronization and Cell Cycle Analysis

Cellular synchronization at the G1/S boundary was performed using thymidine (Sigma-Aldrich, St. Louis, MO, USA) following the method proposed by Chen and Deng [18]. After the cell cycle resumed, cells were collected at 0, 2, 4, 8, 16, 24, and 48 h for further analyses.

Cells seeded for the synchronization assay were collected and fixed overnight at 4 °C in 70% ice-cold ethanol. After washing with ice-cold PBS, cells were stained with a 25 μg/mL propidium iodide solution in the presence of 200 μg/mL RNase A at 37 °C for 45 min in the dark. The cell cycle distribution was determined by flow cytometry in a Beckman Coulter Gallios Flow cytometer in the General Research Services SGIker of the UPV/EHU, with a total acquisition of 10,000 events. The percentage of cells in different phases of the cell cycle was analyzed using Summit 4.3 software (Dako, Hovedstaden, Denmark).

### 2.8. Statistical Analyses

Quantitative data are expressed as the mean ± standard deviation of at least 3 experiments using a Microsoft Office 2010 Excel spreadsheet. The significance of the differences between the means was determined using two-tailed Student’s *t*-tests. Differences were considered statistically significant when *p* < 0.05.

## 3. Results

We used PCR techniques to detect mRNAs of the PON genes, quantitative Western blot to quantify the proteins, and immunofluorescence and confocal microscopy to analyze their subcellular localization. In order to optimize the methodologies employed, and due to the reduced sample volume of human fresh granulosa cells, we used the stable hepatocarcinoma cell line HepG2, with high PON expression [19,20] along with the human ovary cell line COV434 [21].

### 3.1. Detection of PON1, PON2, and PON3 mRNAs by RT-PCR

The optimization of detection by RT-PCR involved testing with different reaction mixtures, hybridization temperatures, and amplification cycles (data not shown). The final conditions of the PCR are described in the Section 2.

**Figure 1:**
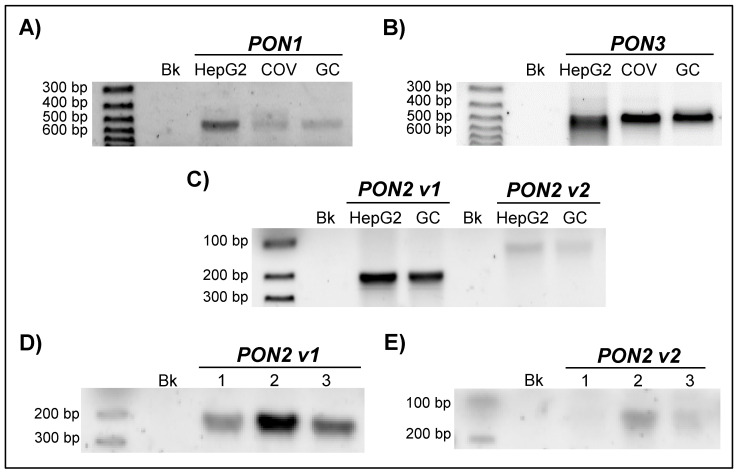
Detection of mRNA of (**A**) PON1, (**B**) PON3, and (**C**–**E**) PON2 variants (v1 and v2) in HepG2, COV434 (COV), and granulosa cells (GC). Bk, blank; 1–3, different samples of COV434.

The amplification of the PON transcripts with specific primers for each of the family members resulted in representative fragments of each transcript (PON1, PON2, and PON3). The primers used for the amplification of the PON1 mRNA were specifically attached to the sequence between exon 1 and exon 4 of the gene, amplifying a 527 bp fragment. This specific fragment was detected in all three cell types studied (Figure 1A). In the case of PON3, the PCR used for its detection allowed for the amplification of a 476 bp sequence between exon 2 and exon 6 of the PON3 gene. It was also detected in all three cell types (Figure 1B).

The PON2 gene is transcribed into different transcripts by alternative splicing [22]. Two main variants, v1 (long) and v2 (short), encode two protein isoforms with lengths of 342 and 354 amino acids, respectively. We designed a PCR protocol capable of differentiating both variants. The cDNA amplification obtained from mRNAs resulting from PON2 transcription was also positive for all three cell types, achieving fragments of 209 bp (v1) and 127 bp (v2) (Figure 1C–E).

### 3.2. Quantification of PON1, PON2, and PON3 by Western Blot

**Figure 2:**
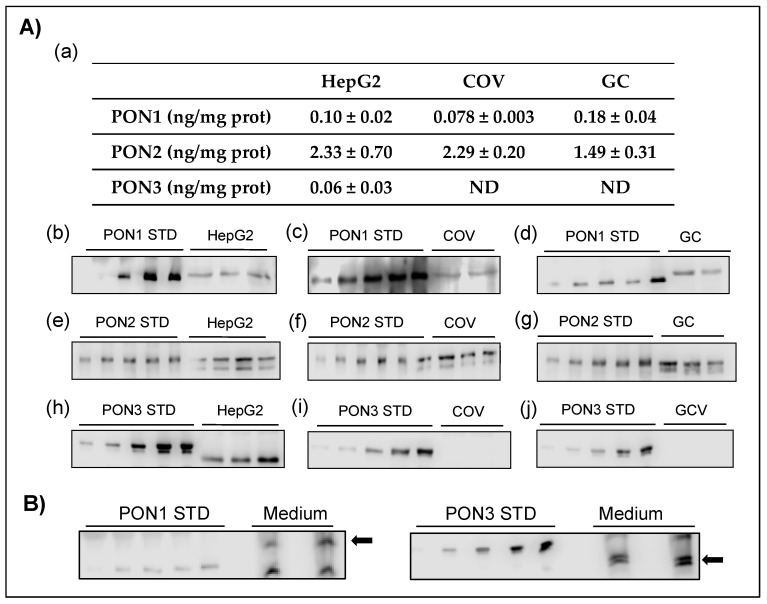
Expression of (**A**) PON1, PON2, and PON3 proteins in HepG2, COV434 (COV), and granulosa cells (GC), and (**B**) PON1 and PON3 proteins in the conditioned medium of granulosa cells after 48 h of incubation. (**a**) The table represents mean values ± SE of the amounts of quantified PON1, PON2, and PON3 in the different cell types. PON3 was not detected in COV434 or granulosa cells. STD lanes indicate increasing concentrations of the corresponding commercially available standard human recombinant protein from which the standard curves were derived. Arrows indicate the positions of PON proteins. ND, not detected.

The protein expression of the components of the PON family was analyzed by quantitative Western blot, using standard curves with human recombinant commercially available proteins (Figure 2).

PON1 and PON2 were detected in HepG2, COV434, and granulosa cells. However, intracellular PON3 was detected only in HepG2 (Figure 2(Aa)). In the case of PON1, a single band corresponding to an apparent 43 kDa molecular weight protein was observed (Figure 2(Ab–Ad)). For this member of the PON family, the concentrations in HepG2 and granulosa cells were similar (Figure 2(Aa)). For PON2, there were two bands corresponding to proteins with apparent molecular weights close to 40 kD (Figure 2(Ae–Ag)), which is in agreement with the alternative splicing process mentioned above. This process results in two isoforms of the same protein, which differ by less than 3 kDa, according to their amino acid sequences [23]. Both variants of the same protein were distinguished in the different cell types studied, indicating the expression of both variants. Since PON2 is an intracellular protein, its concentrations were higher than those of PON1 (Figure 2(Aa)). The concentration found in HepG2 and COV434 appeared to be greater than that determined in granulosa cells, although the differences were not statistically significant.

With respect to PON3, the protein band in HepG2 was clearly visible (Figure 2(Ah)). However, PON3 was not detected by immunoblotting in COV434 or granulosa cells (Figure 2Ai,Aj). This could be due to the low intracellular concentration of this secretion protein, which makes it undetectable by Western blot.

We decided to analyze the secreted PON1 and PON3 in the conditioned medium after 48 h of cell incubation. Firstly, the medium was enriched with minority proteins by partially reducing the concentration of more abundant proteins by affinity chromatography. When analyzing the conditioned medium of granulosa cells, a band corresponding to PON3 was detected by Western blot (Figure 2B). Secreted PON1 was also detected in the medium, resulting in a band of an apparent 43 kDa molecular weight (Figure 2B). The concentration was about nine-fold higher than that of PON3.

### 3.3. Immunocytochemical Detection of PON1, PON2, and PON3

**Figure 3:**
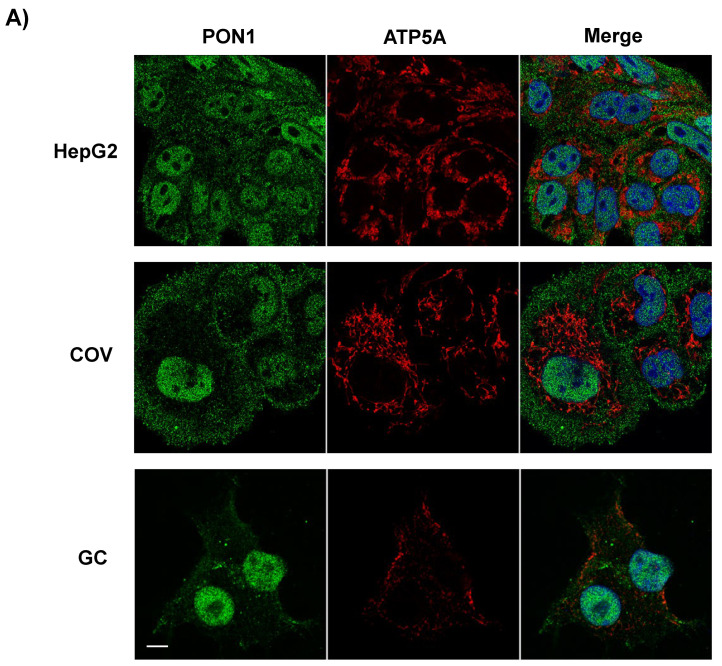

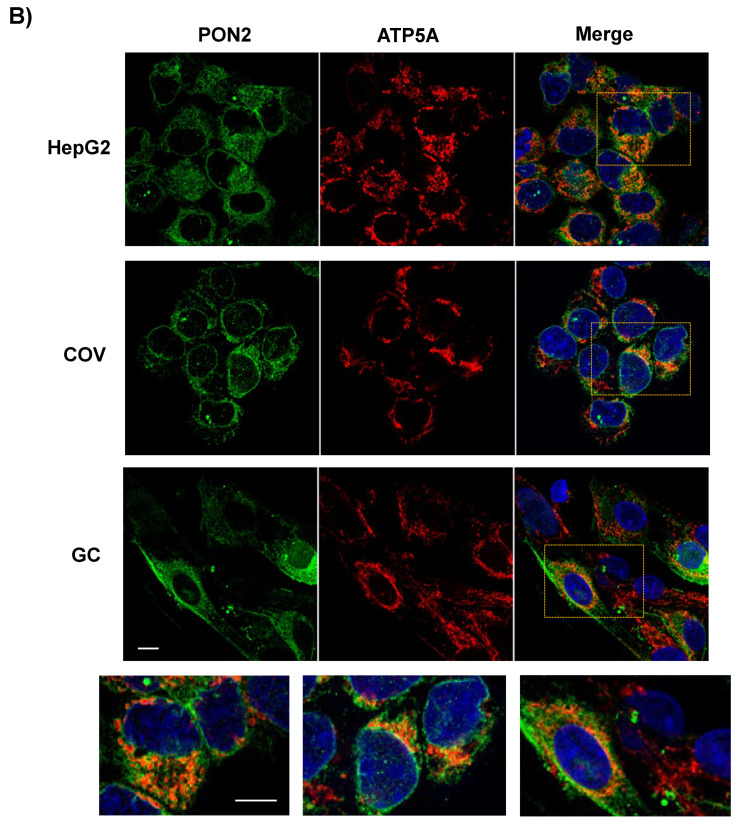

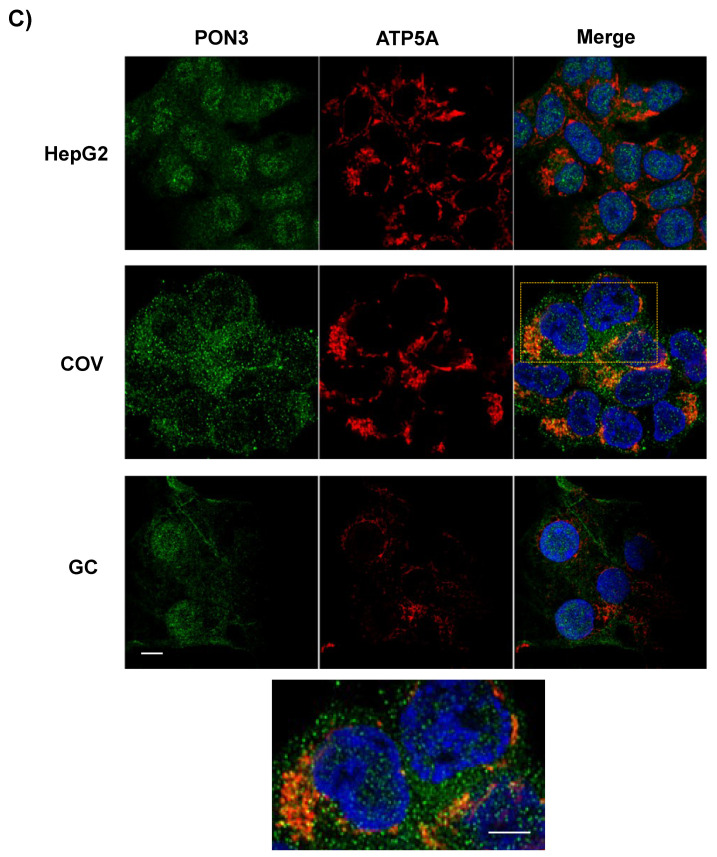
Intracellular distribution of (**A**) PON1, (**B**) PON2, and (**C**) PON3 in HepG2, COV434 (COV), and granulosa cells (GC). PON proteins appear in green and mitochondria in red. The nuclei were stained with DAPI (blue). Cells were examined by confocal microscopy. The white scale bar corresponds to 5 µm. (**B**,**C**) Partial magnification of the areas from HepG2, COV434, and HepG2 indicated in the orange boxes is shown.

Studies of protein intracellular distribution by immunofluorescence and confocal microscopy showed different locations of PON1, PON2, and PON3 (Figure 3). In HepG2, COV434, and granulosa cells, PON1 was localized diffusely in the cytoplasm and apparently excluded from the mitochondria. The nuclear staining was remarkable and avoided the nucleolus and nuclear membrane spaces (Figure 3A). PON2 was distributed in the cytoplasm with a punctate pattern and was largely associated with the mitochondria. A perinuclear enrichment was also appreciated in HepG2 and COV434, probably with a partial nuclear membrane localization (Figure 3B). PON3 showed a pattern of intracellular localization similar to PON1, but the presence in mitochondria could not be discarded in COV434 (Figure 3C).

### 3.4. Synchronization of the Cell Cycle and Subcellular Localization of PON1

**Figure 4:**
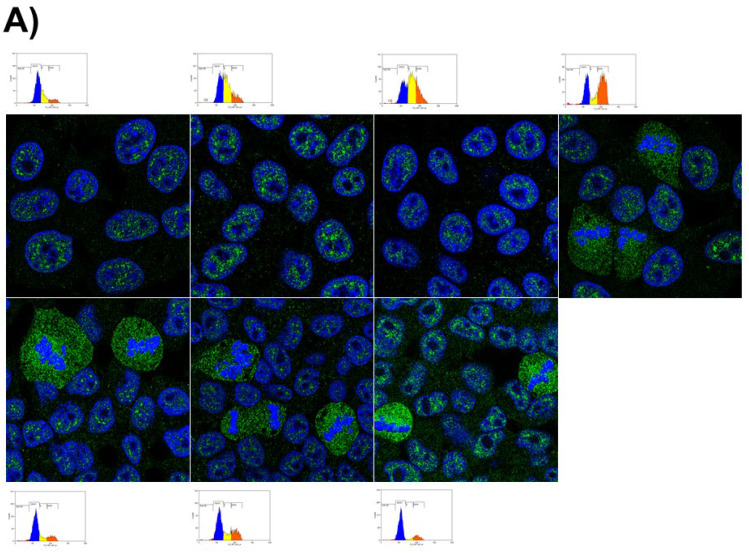

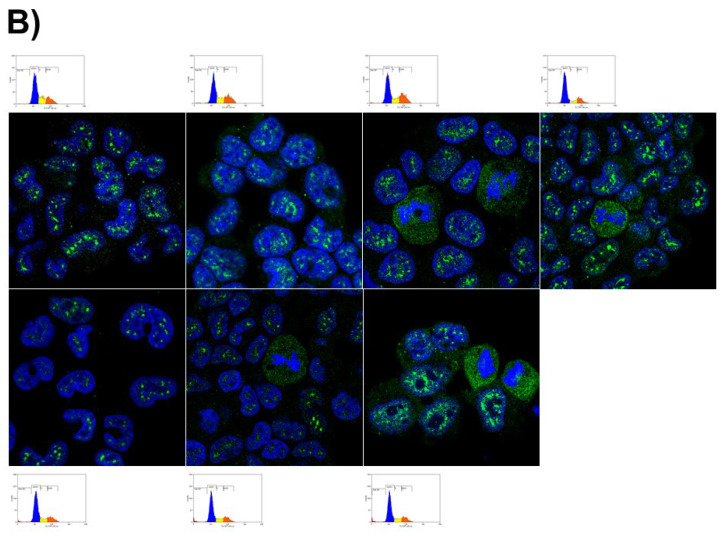
Intracellular distribution of PON1 in (**A**) HepG2 and (**B**) COV434 after cell cycle synchronization. Cells were synchronized and fixed or released for up to 48 h and then fixed. The PON proteins appear in green. Nuclei were stained with DAPI (blue). Confocal images were taken at the indicated times after the cell cycle resumed. Small inserted graphs represent cell distribution along the different cell cycle steps.

PON1 rendered an intriguing nuclear localization, and as a result, a study of the dependence of the subcellular localization on the cell cycle was carried out. HepG2 and COV434 cell lines were subjected to a double-thymidine cell cycle blockade. Cell cycle blocking with thymidine-synchronized cells at the G1/S boundary can be seen in the graphs at time 0 (Figure 4). At this time, the distribution of PON1 was established in discrete granules in the nucleus. As time progressed after the cell cycle resumed, some more extranuclear staining could be appreciated. There was heavy staining of the whole dividing cell, excluding the condensed chromosomes (Figure 4).

## 4. Discussion

This study provides evidence, for the first time, that human ovarian granulosa cells express the genes PON1, PON2, and PON3 both at the mRNA and protein levels. To date, and to the best of our knowledge, local synthesis of these proteins in ovarian reproductive cells has not been reported in women. The PON2 and PON3 mRNAs were reported to be expressed in granulosa cells in dairy cows, but PON1 mRNA could not be detected [24]. The authors proposed that the activity of PON1 measured in follicular fluid could be due to the PON1 protein associated with plasma HDL that is transferred from the blood to the follicular fluid. Furthermore, Marsillach et al. previously reported the presence of PON1, PON2, and PON3 proteins in the ovaries of mice, measured by immunohistochemical analysis of ovarian tissue sections [25]. The authors observed no colocalization with Apo A-I, suggesting local synthesis. They also observed fairly weak staining of PON proteins in granulosa cells. Based on our results on the characterization of PON1 and PON3 transcripts in human granulosa cells, the quantification of PON1 protein inside the cells and of PON1 and PON3 proteins in the incubation medium clearly indicated that both proteins are synthesized by ovarian cells and are secreted to the medium independently of HDL of serum origin.

Regarding the function of these proteins in the follicle, antioxidants are necessary to maintain harmless levels of reactive oxygen species in the oocyte microenvironment, which is essential for the production of a healthy embryo [26]. Paraoxonases may have a role in this protection, as the enzyme activity of PON1 is positively related to the quality of the embryo and the number of blastomeres in women undergoing in vitro fertilization [27]. Similarly, the exogenous addition of recombinant PON1 during the in vitro maturation of bovine oocytes enhanced embryonic development [28]. Proteomic analysis recently revealed the upregulation of PON1 in spontaneous in vitro hatching human blastocysts, suggesting that PON1 is implicated in the hatching process and is critical for blastocyst implantation in the uterus [29]. In another proteomic analysis, PON3 was overexpressed in media from blastocysts with subsequent implantation, thus pointing to a similar role of PON3 in implantation [30].

In the current study, PON1 was localized by immunocytochemistry in the cytosol and, surprisingly, in the nucleus in the three cell types studied, HepG2, COV434, and, at particularly high concentrations, in granulosa cells. These results are consistent with a work in which the intracellular paraoxonase activity of PON1 was detected mostly in nuclear fractions and to a lesser extent in microsomal fractions of the human liver [31]. The PON1 activity distribution was different in the liver from rats, where it was exclusively localized in the microsomal fraction.

The technique of cell synchronization is used to study the molecular mechanisms involved in cell cycle progression. The changes we observed for PON1 in the nucleo-cytoplasmic distribution after blocking cells at G1/S suggest that the protein is associated with the cell cycle process. In cancer studies, PON1 overexpression was recently reported to support the metastatic progression of lung cancer by decreasing the G1/S ratio. In contrast, PON1 knocking down caused cells to be arrested, with changes in the intracellular pattern distribution between the nucleus and the cytosol [32]. The role of PON1 expression and subcellular distribution along the cell cycle is an area of research still to be addressed. PON3 is also localized to the nucleus. We used cNLS Mapper software (http://nlsmapper.iab.keio.ac.jp, date of access 21 September 2020) for nuclear localization signal (NLS) prediction in PON1 and PON3 primary structures [33]. The results showed one bipartite NLS in the PON1 sequence (score 4) and another in the PON3 sequence (score 3.1). The software states that scores of 3, 4, or 5 correspond to proteins that are predicted to be localized in both the nucleus and the cytoplasm. In a study of the networks of protein–protein interactions, Huttlin et al. described a strong interaction between nuclear factor Y (NF-Y) and PON1 [34]. These data are consistent with the presence of PON1 in the nucleus. However, further studies are necessary to establish the biological relevance of these findings, particularly in granulosa cells.

Regarding the PON2 protein, we found that it was the most abundant member of the PON family inside the cells. Immunocytochemistry results showed that PON2 had a perinuclear distribution and colocalized with the mitochondria. Considering that the mitochondria are the major source of free radicals, PON2 would protect the ovarian cells from oxidative stress. A study carried out by Horke et al. demonstrated the presence of PON2 in the nuclear envelope and the endoplasmic reticulum in vascular cells, which is consistent with the perinuclear distribution we have found [23]. The proteins in the endoplasmic reticulum are also targets of oxidative stress, so the action of PON2 in this organelle would participate in reducing ROS levels. It was suggested that each of the two isoforms of PON2 may have a different location inside the cell [23], which would be compatible with their distribution in both the mitochondria and the endoplasmic reticulum.

In the present study, the confocal microscopy results also pointed to mitochondrial localization for PON3 in COV434. In this sense, in embryonic kidney HEK293 cells, the PON3 isoform overlapped with a mitochondrial marker, reinforcing the association with this organelle [35]. The authors also reported that overexpressed PON3 in the cancer cell lines diminished the intramitochondrial formation of superoxide anions, suggesting a direct interaction with the Q10 coenzyme.

The current work is an observational study that provides descriptive data, and no functional assays were performed. We are conscious that superovulation treatment may have effects on the gene expression in human granulosa cells [36] and perhaps on the intracellular protein sorting. Further research manipulating the expression of PON proteins in granulosa cultures would reveal physiological roles in these cells. In vivo correlations of PON expression and localization in granulosa cells from women with different causes of infertility (male, female, couple, environmental, or unexplained factors) with fertilization parameters would contribute to understanding the relevance of these proteins in female fertility.

## 5. Conclusions

For the first time, we showed that human ovarian granulosa cells express PON1, PON2, and PON3 genes at both the transcriptional and protein levels. The PON proteins were differentially localized within the cells. This work represents the basis for studies focused on the functions of these proteins in granulosa cells, which will contribute to a better understanding of the reproduction process.

## Figures and Tables

**Table 1:** Sequences of the primers designed for PCR.

Gene Target	Forward (5′ → 3′)	Reverse (5′ → 3′)
PON1	CCAGTCTTCTTACCAAACACGA	GGGTTGAAGCTCTTTATTCCA
PON2		
Variant 1	TTAGTGTGGGTCTAAAATTCCC	TGGGTGGTTTACAACAAAGAG
Variant 2	TACTAATGATGGATCTAAAAGAAGAAAAAC	GTATTCTTGAATTCGTCTATG
PON3		
Set 1	AGATGTTCCTGGCGTTTAG	ACCTCCCTTGGGCTGTAGAA
Set 2	GAGAAGTGGAGCCAGTAGA	ATTTCTAGCGCTTGTGCCCT

**Table 2:** Conditions of the amplification of *PON1*, *PON2* variants, and *PON3* transcription products by PCR.

Gene Target		Denaturing	Hybridization	Extension	No. of Cycles
***PON1***	Temperature	94 °C	58.6 °C	72 °C	40
	Time	1 min	1 min	1 min	
***PON2* variant 1**	Temperature	94 °C	52.5 °C	72 °C	30
	Time	1 min	1 min	1 min	
***PON2* variant 2**	Temperature	94 °C	54.5 °C	72 °C	30
	Time	1 min	1 min	1 min	
***PON3***					
PCR1	Temperature	94 °C	54 °C	72 °C	30
	Time	1 min	1 min	1 min	
PCR2	Temperature	94 °C	52 °C	72 °C	30
	Time	1 min	1 min	1 min	

## Data Availability

Data is contained within the article.
